# The Effect of Wearable Tracking Devices on Cardiorespiratory Fitness Among Inactive Adults: Crossover Study

**DOI:** 10.2196/31501

**Published:** 2022-03-15

**Authors:** Lisbeth Hoejkjaer Larsen, Maja Hedegaard Lauritzen, Mikkel Sinkjaer, Troels W Kjaer

**Affiliations:** 1 Department of Neurology Zealand University Hospital Roskilde Denmark; 2 Faculty of Health and Medical Sciences University of Copenhagen Copenhagen Denmark

**Keywords:** activity tracking, cardiorespiratory fitness, mHealth, mobile health, motivation, physical activity, self-monitoring, wearable, cardio, fitness, cardiorespiratory, behavior change

## Abstract

**Background:**

Modern lifestyle is associated with a high prevalence of physical inactivity.

**Objective:**

This study aims to investigate the effect of a wearable tracking device on cardiorespiratory fitness among inactive adults and to explore if personal characteristics and health outcomes can predict adoption of the device.

**Methods:**

In total, 62 inactive adults were recruited for this study. A control period (4 weeks) was followed by an intervention period (8 weeks) where participants were instructed to register and follow their physical activity (PA) behavior on a wrist-worn tracking device. Data collected included estimated cardiorespiratory fitness, body composition, blood pressure, perceived stress levels, and self-reported adoption of using the tracking device.

**Results:**

In total, 50 participants completed the study (mean age 48, SD 13 years, 84% women). Relative to the control period, participants increased cardiorespiratory fitness by 1.52 mL/kg/minute (95% CI 0.82-2.22; *P*<.001), self-reported PA by 140 minutes per week (95% CI 93.3-187.1; *P*<.001), daily step count by 982 (95% CI 492-1471; *P*<.001), and participants’ fat percentage decreased by 0.48% (95% CI –0.84 to –0.13; *P*=.009). No difference was observed in blood pressure (systolic: 95% CI –2.16 to 3.57, *P*=.63; diastolic: 95% CI –0.70 to 2.55; *P*=.27) or perceived stress (95% CI –0.86 to 1.78; *P*=.49). No associations were found between adoption of the wearable tracking device and age, gender, personality, or education. However, participants with a low perceived stress at baseline were more likely to rate the use of a wearable tracking device highly motivating.

**Conclusions:**

Tracking health behavior using a wearable tracking device increases PA resulting in an improved cardiorespiratory fitness among inactive adults.

## Introduction

In the Western world, physical inactivity and sedentary behavior are increasing and accordingly, so are health-related problems and health care costs. Global Health Observatory data estimate that 37% of the adult population in high-income countries is insufficiently physically active [1]. In Denmark, 29% of the adult population report that they do not meet the World Health Organization’s minimum recommendation for physical activity (PA), and of them, 71% want to be more physically active [2,3]. Starting and maintaining a physically active life is a great challenge for many people.

Wearable tracking devices (WTDs) have been suggested to support and motivate to a physical active behavior [4]. WTDs are small wearable computers with sensors that monitor different health-related parameters such as steps, physical intensity minutes, and heartbeat continuously under real-life conditions. Despite the promising features embedded in WTDs the results are mixed from studies investigating the effect of increasing PA with the use of these devices. Three recent reviews conclude that the use of a WTD improves daily step counts regardless of age, sex, and health status, but less consensus is found regarding the effect on moderate to vigorous PA (MVPA) [5-7]. Few studies have investigated the effect on cardiorespiratory fitness (CRF) despite low CRF has been reported to be a more powerful predictor of health issues than, for instance, inactivity [8,9]. Discrepancy exists between the few studies that have evaluated the effect on CRF after a WTD intervention [10-14]. The existing studies were all carried out at least 5 years ago and thereby conducted with older devices. Because a low CRF constitute a separate risk factor, the effect of utilizing a modern WTD on CRF is relevant to clarify [15]. In addition, not all individuals exhibit the same tendency for using a WTD, and recent studies suggest that individual differences may play a role in the adoption of using a WTD [4,16,17]. For instance, a study found that behavioral intentions to use a WTD is affected by personality traits, age, computer self-efficacy, and prior PA [18].

The purpose of this study was to investigate the effect of using a modern WTD on CRF and the relationships between the adoption of using a WTD and personal characteristics and health outcomes.

## Methods

### Participants

Participants were recruited from Naestved city, Denmark, through local advertisements in media (newspaper, television, radio, and the internet). Participants were required to be at least 18 years of age and to own a smartphone or tablet device. Only inactive participants who reported exercising less than the recommended 150 minutes per week [3] were eligible for the study.

The primary outcome was CRF, and the minimal difference of interest in Vo_2_max was 2 O_2_/kg/minute. With a significance level of *P*=.05 (2-sided), a total number of 56 participants should be included using an SD of 4.5 O_2_/kg/minute to obtain a 90% power to detect the minimal difference of interest. The SD was based on the difference between 2 measures for the same participant obtained in a feasibility study [19]. Allowing for an attrition rate of 10%, 62 participants should be included.

### Ethics Approval

The study was approved by the ethics Committee of Region Zealand (protocol SJ-780) and was performed in accordance with the Declaration of Helsinki.

### Experimental Protocol

**Figure 1:**
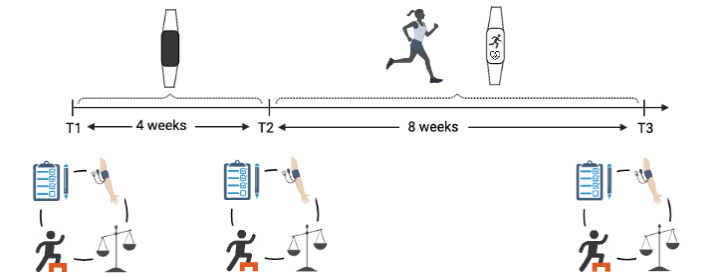
Experimental protocol (Created with BioRender.com).

Participants attended three test days: a baseline test day (T1) followed by 4 weeks of observation, a second test day (T2) followed by 8 weeks of intervention, and a third test day (T3) at the end of the intervention (Figure 1).

A WTD (Garmin Vivosmart 4, CE marking) was handed out to all participants at T1. This WTD detects movement and heart rate via an embedded triaxial accelerometer, optical photoplethysmography signals, and associated algorithms. It automatically records intensity and the duration of different activity patterns, and estimates active kilocalories. It also attempts to obtain an objective estimate of stress on the basis of the root mean square of successive R-R intervals [20] and of sleep staging through a combination of accelerometer and photoplethysmography [21]. Participants were instructed to download a mobile app called “Garmin Connect” and set up a user account. Participants were required to wear the device on their wrist for the entire period of approximately 12 weeks. Between T1 and T2, participants were instructed to continue their usual lifestyle. During these first 4 weeks participants were asked to refrain from looking at their data. The screen on the WTD was customized to display only the clock. After 4 weeks of observation, participants had an extended introduction to the WTD and the accompanied Garmin Connect mobile app in which they were able to follow their health behaviors. Between T2 and T3, participants were instructed to increase their PA level to a least 150 intensity minutes per week and to use the WTD to register and follow their PA behavior. The WTD was installed in collaboration with participants and the number and type of received notifications were individualized in accordance with the participants’ own wish. All participants were instructed to upload their data via Garmin Connect at least once a week.

### Outcome Measures

Personal characteristics of participants were collected from a survey during T1 and included age, gender, years of education, family status, and smoking. The survey also included three validated questionnaires:

1. The NEO Five Factor Inventory questionnaire (NEO-FFI-3): this questionnaire consists of 60 items and provides a measure of the 5 domains of personality (neuroticism, extraversion, openness, agreeableness, and conscientiousness). The internal consistency for the NEO-FFI-3 ranges from 0.79 to 0.86 [22].
2. The Nordic Physical Activity Questionnaire-short is a 2-item questionnaire and provides a measure of MVPA in minutes per week (Spearman ρ=0.33 between self-reported and objectively measured PA levels) [23].
3. The Perceived Stress Scale [24] assess subjective stress levels and comprises 10 items. Scores range from 0 to 40, with higher composite scores indicating greater levels of perceived stress.

The latter 2 questionnaires were also completed at T2 and T3 to explore changes in self-reported measures. At T3, the participants were also asked to evaluate the motivational impact of using a WTD (self-reported adoption) on a 5 ordered level ranging from 4=highly motivated to 0=not helpful. A short web-based survey was sent out 6 months post study participation with questions of current PA behavior.

Height was measured with a stadiometer (Leicester portable height measure Tanita HR 001). Body composition, including body weight (in kg), fat percentage, and skeletal muscle (in kg) were assessed through bioelectrical impedance analysis using the monitor Tanita DC 430 SMA [25]. Blood pressure was monitored in a sitting position with an automated oscillatory device (Omron M3) after the participant had rested for 5 min. The lowest mean arterial pressure of 3 readings was used. Finally, the new step test was conducted and used to estimate participants CRF [26]. The step test is a progressive test based on the principle that the energy cost of stepping with a known step height and pace is relatively independent of age, gender, and training status. The test starts with a slow stepping frequency (0.2 steps per second), which increases gradually to a very fast stepping frequency (0.8 steps per second) after 6 minutes. The CRF is estimated on the basis of the stopping time; that is, the time when the pace can no longer be followed.

The following health parameters were exported from the WTD: steps, MVPA, active kilocalories, resting heart rate (HR), stress scores, and total sleep time. Compliance of wearing the WTD is important for the accuracy of the measurements and was calculated on the basis of automatically registered HR measures relative to the study duration. More than 10 minutes of continuous missing HR data were registered as missing data. Thus, the percentage of available HR data was used as a proxy for the percentage of time participants wore the WTD.

### Analysis and Statistics

Baseline characteristics are presented as mean (SD) or n (%) values. All data were imported to MATLAB (R2017_b) for analysis. Statistical processing of the data was carried out with R statistical program (RStudio; version 1.2.5033, packages: nlme, clubSandwich). For analyses of the primary outcome (CRF), we used a linear mixed model for repeated measures over time to analyze the difference among the 3 test days. Time was considered a fixed effect, and participants was considered a random effect, and the maximum likelihood method was applied. A similar procedure was used for secondary outcomes such as body composition, blood pressure, and self-reported PA. For nonnormally distributed variables, cluster-robust variance estimators with “CR2” adjustment were applied [27].

Daily measures obtained with the WTD included step count, MVPA active kilocalories, resting HR, stress score, and total sleep time. A calibration period for the WTD was recommended; hence, the first 7 days in the control period were excluded from further analysis. The mean of each measure was calculated for the control and the intervention period, respectively. A 2-tailed Student *t* test was used to test for differences in the normally distributed variables. Objectively measured MVPA was compared with self-reported PA with a Pearson correlation analysis.

The influence of personal characteristics and health outcomes on the adoption of using a WTD was explored by fitting a linear model. The adoption of WTD was based on the participants subjective evaluation of the motivational impact of using a WTD at T3. This response variable was chosen as we believe perceived motivation is the best prediction of future use. The following baseline variables were included inspired by previous studies [16,17,28]: personal characteristics (age, gender, education, and personality) and current individual health status at T1 (BMI, fat percentage, CRF, self-reported PA, perceived stress, daily step count, and active kilocalories). Moreover, changes in health outcomes at T3 (changes in CRF, fat percentage, BMI, self-reported PA, perceived stress, daily step count, and active kilocalories) were also included in the analysis to explore if improvements of health parameters were specifically related to adoption of WTD. One subject was excluded from the analysis owing to 36% of missing values. Other observations with missing values were imputed using k-nearest neighbors. All the predictor variables were standardized, such that they have a mean of 0 and SD of 1. Feature selection was applied using Partial Least Squares [29] to reduce the effect of variables with multicollinearity. The number of significant components was then determined by The Weight Randomized Test [30]. For the significant number of components, The Variable Importance in Projection was calculated to select variables with a score greater than 1 for further analysis [31]. Principal components analysis was performed on health parameters to secure independents. The score from the principal components analysis was used as predictor variables for the linear regression model. To obtain a model solely on the basis of significant effects, stepwise regression was performed for the linear model.

## Results

**Figure 2:**
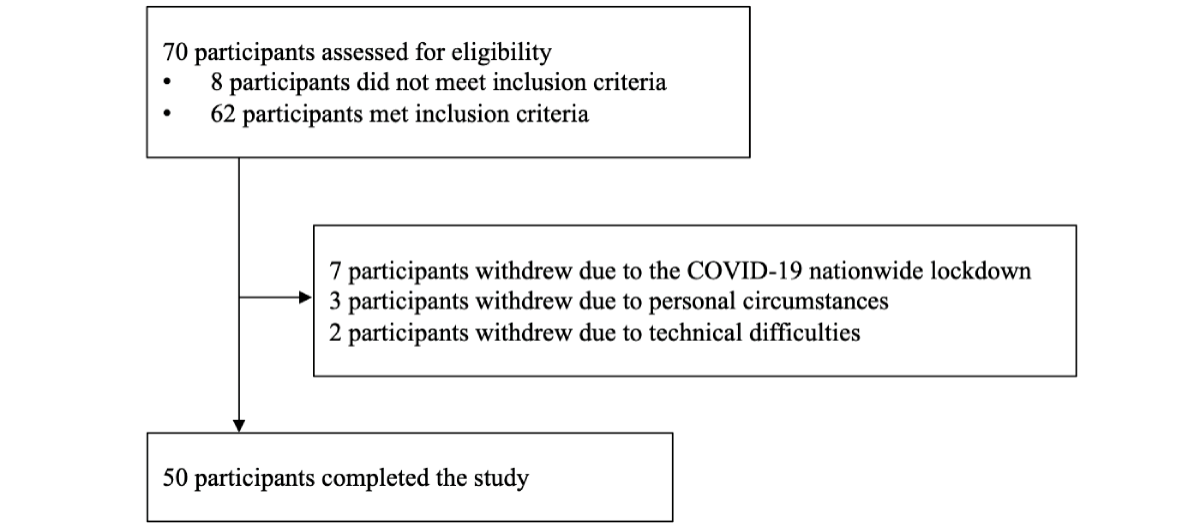
Flow diagram of participant inclusion.

Participants’ baseline characteristics are shown in Table 1. Data collection was initiated in October 2019 and ended 1 year later. In total, 16 participants were paused in March 2020 owing to a nationwide COVID-19 lockdown, of whom 7 completely withdrew from the study, 2 completed T3 on the internet, and 7 restarted their intervention period after the lockdown end of April 2020. The incidence of COVID-19 cases increased during fall 2020, which led to gradual restrictions on physical training facilities and size of participation in teams sport and group exercises toward the end of the study period. In addition, during the data collection, 3 participants withdrew owing to personal circumstances (not related to the study or the COVID-19 pandemic), and 2 withdrew owing to technical difficulties. A flow diagram is presented in Figure 2.

**Table 1:** Participants’ baseline characteristics.

Baseline characteristics	All participants (N=62)	Analyzed population (n=50)
Age (years), mean (SD)	50 (14)	48 (13)
Female, n (%)	51 (82)	42 (84)
Male, n (%)	11 (18)	8 (16)
Education (years), mean (SD)	2 (14)	2 (14)
Married or living together, n (%)	37 (60)	32 (64)
Children at home under 16 years of age, n (%)	22 (35)	20 (40)
Current smoker, n (%)	7 (11)	3 (6)
Neuroticism, mean (SD)	44 (10)	44 (10)
Extraversion, mean (SD)	51 (11)	52 (11)
Openness, mean (SD)	54 (9)	53 (9)
Agreeableness, mean (SD)	57 (10)	58 (11)
Conscientiousness, mean (SD)	56 (10)	56 (11)

The duration of the control and intervention period were 30 (SD 5) days and 61 (SD 6) days, respectively. The results of objective and self-reported health parameters are shown in Table 2. One participant did not conduct the step test at T1 owing to guidelines for marked elevated hypertension (BP>180/105 mm Hg) [32]. Because of the COVID-19 lockdown, 4 patients solely completed the web-based survey on one of the test days with no measures of body weight, BP, or CRF. Moreover, one participant did not conduct the step test either at T2 and T3 owing to hip pain aggravated by the step test, and 4 participants did not conduct the final step test at T3 owing to temporary knee pain and dizziness, respectively. Finally, 2 participants had their antihypertensive medication adjusted during the study (not related to study activities) and were therefore excluded from the BP analysis.

**Table 2:** Results of the linear mixed model on objective and self-reported health parameters.

Parameter (n)		Estimated mean (SE)	*P* value	95% CI
**Cardiorespiratory fitness** **(mL/kg/minute)** **(50)**				
	Intercept T1	26.42 (0.85)		24.75 to 28.09
	Difference from T1 to T2	0.89 (0.35)	.01^a^	0.21 to 1.57
	Difference from T2 to T3	1.52 (0.36)	<.001^a^	0.82 to 2.22
**BMI (kg/m^2^) (50)**				
	Intercept T1	27.88 (0.82)		26.26 to 29.49
	Difference from T1 to T2	–0.02 (0.06)	.81	–0.14 to 0.11
	Difference from T2 to T3	–0.12 (0.08)	.13	–0.29 to 0.04
**Fat percentage (%) (50)**				
	Intercept T1	32.63 (1.00)		30.67 to 34.59
	Difference from T1 to T2	0.05 (0.15)	.72	–0.24 to 0.34
	Difference from T2 to T3	–0.48 (0.18)	.009^a^	–0.84 to –0.13
**Muscle mass (kg) (50)**				
	Intercept T1	50.50 (1.39)		47.77 to 53.23
	Difference from T1 to T2	0.03 (0.12)	.83	–0.20 to 0.25
	Difference from T2 to T3	0.11 (0.16)	.51	–0.21 to 0.42
**Systolic blood pressure (mm Hg) (48)**				
	Intercept T1	124.69 (2.51)		119.75 to 129.62
	Difference from T1 to T2	–2.22 (1.37)	.11	–4.92 to 0.47
	Difference from T2 to T3	0.71 (1.46)	.63	–2.16 to 3.57
**Diastolic blood pressure (mm Hg) (48)**				
	Intercept T1	81.23 (1.48)		78.31 to 84.15
	Difference from T1 to T2	–1.70 (0.85)	.047^a^	–3.36 to –0.04
	Difference from T2 to T3	0.93 (0.83)	.27	–0.70 to 2.55
**Moderate to vigorous physical activity (minutes per week) (49)**				
	Intercept T1	94.18 (11.6)		70.9 to 117.4
	Difference from T1 to T2	7.24 (14.9)	.63	–22.8 to 37.3
	Difference from T2 to T3	140.19 (23.3)	<.001^a^	93.3 to 187.1
	Difference from T2 to 6 months	58.62 (23.6)	.02^a^	10.6 to 106.6
	Difference from T3 to 6 months	–81.6 (29.2)	.008^a^	–140 to –22.6
**Perceived Stress Scale score (50)**				
	Intercept T1	13.54 (0.87)		11.79 to 15.29
	Difference from T1 to T2	–3.14 (0.57)	<.001^a^	–4.29 to –1.99
	Difference from T2 to T3	0.46 (0.66)	.49	–0.86 to 1.78

^a^Values are significant at *P*<.05.

A significant increase in CRF of 0.89 mL/kg/minute (*P*=.01) was observed already at T2. CRF increased further during the intervention period with 1.52 mL/kg/minute (*P*<.001; see Table 2). No change was observed in BMI and muscle mass, while the fat percentage decreased from T2 to T3 by 0.48% (*P*=.009). No change was observed in systolic BP, while diastolic BP decreased with 1.7 mm Hg (*P*=.047) during the control period with no further change during the intervention period. Perceived stress decreased from T1 to T2, with a reduction of 3.14 in the Perceived Stress Scale score (*P*<.001), while no change was observed between T2 and T3. The result from the self-reported PA questionnaire was omitted for one participant owing to an incorrect completion of the questionnaire (the participant reported a higher number of vigorous active minutes per week than total MVPA minutes per week). Self-reported PA behavior from the remaining 49 participants was unchanged between T1 and T2 and increased during the intervention period with 140 minutes per week (*P*<.001). In total, 26 (50%) participants replied to the 6-month follow-up survey from which it appeared that self-reported exercise behavior was significantly higher than that before the intervention (59 minutes, *P*=.02), but also significantly lower than that at the end of the intervention (82 minutes, *P*=.008). Of note, a distinct variation in PA behavior among participants was observed after 6 months in the associated 95% CIs (see Table 2).

Throughout the intervention period, the participants wore the WTD device 94% (SD 5%) of the time. The daily step count increased by 982 steps per day (*P*<.001) from the control to the intervention period, objectively measured MVPA by 107 minutes per week (*P*<.001), and active kilocalories by 180 kilocalories per day (*P*<.001). Resting HR decreased from 58 to 57 beats per minute from the control to the intervention period (*P*=.002), while no change was observed in daily stress scores or total sleep time (Table 3). Objectively measured MVPA significantly correlated with self-reported PA in the intervention period, where participants were encouraged to register PA on the WTD (*r*=0.38, *P*=.008). A similar correlation was not observed in the control period, where participants were instructed to refrain from actively using the WTD (*r*=–0.03, *P*=.86).

**Table 3:** Average measures obtained with wearable tracking device use (stress scores ranged from 0=low to 100=high and are based on the root mean square of successive R-R intervals).

Wearable tracking device measures	Control period, mean (SD)	Intervention period, mean (SD)	*P* value	Mean difference (95% CI)
Steps per day	8196 (2446)	9178 (2735)	<.001^a^	982 (492 to 1471)
Moderate to vigorous physical activity per week	86 (187)	193 (190)	<.001^a^	107 (74 to 140)
Active kilocalories per day	332 (222)	512 (257)	<.001^a^	180 (129 to 231)
Resting heart rate (beats per minute)	58 (8)	57 (7)	.002^a^	–1 (–1.3 to –0.3)
Stress score per day	31 (9)	31 (6)	.77	0 (–2.2 to 1.6)
Total sleep time per night (hours:minutes)	07:42 (46)	07:49 (42)	.10	00:07 (–1.3 to 14.0)

^a^Values are significant at *P*<.05.

Participants rated the motivational impact of using a WTD on a 5 ordered level ranging from 4=highly motivated to 0=not helpful. In total, 16 participants rated the impact with “4,” 16 participants rated “3,” 9 participants rated “2,” 7 participants rated “1,” and 2 participants did not find the WTD helpful (score=0). The motivational impact of using a WTD (the response variable) and predictor variables (personal characteristics, current individual health status, and health outcomes) revealed (via partial least squares regression) a significant first component after applying the Weight Randomized Test. In this first component, the following variables displayed a Variable Importance in Projection scores of >1: age, baseline perceived stress, BMI, and active kilocalories as well as changes in fat percentage, active kilocalories, step count, and BMI. Principal components analysis was performed for the latter 6 variables. Age, baseline perceived stress, and scores for each principal component were used as predictors in the linear model. After stepwise regression, the final model contained an intercept and the estimated effects of baseline perceived stress and the first principal component (see Table 4). The loadings of the first principal component are shown in Table 5.

**Table 4:** The estimated effects of the linear model.

Parameters	Estimate (SE)	*P* value
Intercept	2.7143 (0.13865)	6.069×10^–24^
Perceived stress at T1	–0.36159 (0.14028)	.01
First principal component	0.38181 (0.088732)	8.716×10^–05^

**Table 5:** The loadings of the first principal component.

Variable	Changes in variables					
	Fat percentage	Step count	BMI	Active kilocalories	T1 active kilocalories	T1 BMI
Loading	–0.542	0.485	–0.485	0.429	–0.229	–0.024

## Discussion

### Principal Findings

Results from this study suggest that the use of WTDs can increase CRF and PA and decrease fat percentage after an intervention of 8 weeks. The primary outcome measurement was CRF, which is less studied in relation to the use of WTDs. In this study, we used an updated, simple, and user-friendly version of a WTD. Previous studies have reported mixed results on CRF after the use of a WTD. Two studies reported significant improvements of 1.8 mL/kg/minute after 6 months [10] and 2 years [13], while 3 studies found no effect after respectively 3 months and 1 year usage of a WTD [11,12,14]. However, these 5 studies were all conducted for more than 5 years ago with quite different activity trackers than currently available. Thus, our finding of an increase in CRF of 1.52 mL/kg/minute after the use of a modern WTD contributes new knowledge in an area of current sparce and mixed results. Improvements in CRF of 3.5 mL/kg/min have been associated with 8% to 35% reductions in mortality [9]. From this perspective, an average increase of 2.4 mL/kg/minute in CRF after the control and intervention period combined suggest a noteworthy health benefit if the participants maintain the increase of PA behavior in future.

We observed an increase of 982 steps per day in the intervention period compared to the control period. Two recent meta-analyses report a positive effect for step count equivalent to approximately 500-627 more steps per day in intervention groups compared to control groups [5,6]. The step count is one of the more validated and accurate measures registered by modern WTDs [33], and the feature is easy for the user to comprehend and track. This could explain the general positive effect.

The effect of WTD on activity minutes is less clear ranging from no significant difference [5] to a mean increase of 75 minutes per week among recent studies [6]. In this study, we observed a convincing increase of 140 minutes per week in self-reported MVPA and of 107 minutes per week in objectively measured MVPA during the intervention period compared to the control period. Self-reporting is known to overestimate PA [34], which may explain part of the discrepancy observed in previous studies and in this study. Moreover, in the literature, some studies obtain the objective measurement of MVPA via validated accelerometers and other studies directly from the commercial WTD, which was carried out in this study. On a WTD, the timely resolution and accuracy of MVPA often depends on user activation of PA. This may explain the lack of correlation between self-reported and objectively measured MVPA in the control period in this study. Thus, part of the discrepancy between current studies, investigating the use of a WTD on MVPA, may relate to application of different methods to assess MVPA.

In this study, a decrease in fat percentage of 0.48% was observed, similar to that reported in a recent randomized controlled trial including 135 adults [35]. In this study, 32 out of 50 (64%) participants were overweight, as assessed from their fat percentage [36]. In line with a previous study investigating 9 months of WTD use, the decrease in fat percentage was more pronounced among overweight participants compared to average or lean participants (0.59% vs 0,16%) [37]. Most previous studies have investigated weight loss and not changes in body composition. There is currently no evidence for the use of WTDs in weight loss among healthy inactive or overweight adults [7,38], and this study confirms this finding.

The effect of the use of a WTD on blood pressure is mixed. A study by Thorndike et al [39] found that systolic BP decreased 3 mm Hg, while diastolic BP did not change after 12 weeks among young medical residents. Another study involving older patients diagnosed with type 2 diabetes showed a significant decrease of 6.7 mm Hg (*P*<.01) in systolic BP and 2.9 mm Hg in diastolic BP (*P*<.05) [40]. In contrast and in line with our results, Finkelstein et al [41] found no improvement in BP among adult employees after the use of a WTD.

The positive effect of PA on stress levels is well documented [42]. However, we solely observed a decrease in perceived stress levels after the control period. The increase in CRF after the control period may have affected stress levels positively. Moreover, the low stress scores (10.4, SD 5.6) observed at T2 seem to represent a ceiling effect, which reduce the potential for improvement.

The effect of the use of a WTD on different health parameters is mixed and is challenged by the fact that many studies are carried out using older versions of WTDs. The technology is developing at a fast pace, and further research is needed to determine the efficacy of the latest devices. Furthermore, current literature evaluating the health benefit of using WTDs differs in study designs, duration of interventions, outcome measures, and participant characteristics.

### The Motivational Impact of Using a WTD

In this study, no associations were found between adoption of the WTD and age, gender, personality, or education (explored with partial least squares regression analysis). This is in contrast to a study from 2018, which indicated that older people (aged >50 years) were less likely to use a WTD, as they perceived the usability as low [18]. It could be speculated that the use of a very simple WTD and the instruction of achieving a clear goal (minimum 150 MVPA minutes per week) positively influenced the perceived utility among participants above 50 years of age in this study. Rupp et al [18] further found that personality traits such as agreeableness, conscientiousness, and extraversion were associated with high intention to use WTDs. However, Attig and Franke [43] could not find these associations, concurrent with our findings. Thus, more research is needed to reveal how personal characteristics are associated with adoption of activity tracking technology.

Adoption of the use of a WTD has also been linked to dynamic variables such as current individual health status. We observed that participants with low perceived stress at T1 were more likely to rate the use of a WTD highly motivating at T3 (Table 4), suggesting that sufficient resources are important for successful adoption. Rupp et al [18] reported that physically active individuals have higher desire to use a WTD, as they are more likely to find such a device motivating [44]. We also observed that participants who were more physically active during the intervention and reduced their fat percentage were more likely to rate the use of a WTD as motivating (Table 5). Similar to this finding, Su et al [28] reported a significantly larger decrease in their primary outcome (change in hemoglobin A_1c_ levels) in active users of an mHealth app compared to that of nonactive users among patients with diabetes.

### Limitations

Some limitations need to be acknowledged. First, we used a commercial WTD with software updates and proprietary algorithms, which only allows access to already processed data and not the raw data. This limits the interpretations of the data since the threshold of different activities are unknown. Second, 28 of 50 participants were tracking their PA behavior via the accompanied Garmin Connect app during the control period, although specifically instructed not to. The information may have affected their behavior and may explain the observed increase in CRF from T1 to T2. However, behavioral outcomes may also be affected simply by the awareness of being monitored [45]. Third, we used an indirect but validated step test to estimate CRF. Fourth, the included sample size was not powered to investigate associations between adoption of a WTD and personal characteristics and health outcomes. Thus, these findings should be interpreted with caution. Fifth, the study was conducted during the COVID-19 pandemic and temporal changes in PA patterns cannot be excluded owing to different home or work patterns and periodic restrictions on physical training facilities. Finally, generalization of our results is limited by the unequal distribution of gender, with 42 women out of 50 participants in our study. However, gender effects are not identified as an influential factor for the use of a WTD in a recent review [16].

### Conclusions

Tracking health behavior using a modern WTD increases PA, leading to an improved cardiorespiratory fitness among inactive adults. The motivational impact of the use of a WTD varied among participants. No associations were observed between personal characteristics (such as age and personality) and self-reported adoption, but participants with a low perceived stress at baseline were more likely to rate the use of a WTD as highly motivating. Furthermore, participants who were more physically active and who reduced their fat percentages during the intervention were also more likely to perceive the WTD as motivating, which suggests that the device contributed significantly to the observed health benefits.

## Abbreviations

BP: blood pressure
CRF: cardiorespiratory fitness
HR: heart rate
MVPA: moderate to vigorous physical activity
NEO-FFI-3: NEO Five Factor Inventory questionnaire
PA: physical activity
WTD: wearable tracking device
